# Yoga intervention and reminder e-mails for reducing cancer-related fatigue - a study protocol of a randomized controlled trial

**DOI:** 10.1186/s40359-019-0339-3

**Published:** 2019-09-18

**Authors:** Teresa Zetzl, Michael Schuler, Agnes Renner, Elisabeth Jentschke, Birgitt van Oorschot

**Affiliations:** 1 Interdisciplinary Center, Palliative Medicine, University Hospital Wuerzburg, Josef-Schneider-Str. 2, 97080 Wuerzburg, Germany; 2 Institute of Clinical Epidemiology and Biometry, University of Wuerzburg, Josef-Schneider-Str. 2, 97070 Wuerzburg, Germany

**Keywords:** Cancer, Fatigue, Yoga, Reminder e-mails, Supportive therapy, Randomized controlled trial

## Abstract

**Background:**

Almost 90% of cancer patients suffer from symptoms of fatigue during treatment. Supporting treatments are increasingly used to alleviate the burden of fatigue. This study examines the short-term and long-term effects of yoga on fatigue and the effect of weekly reminder e-mails on exercise frequency and fatigue symptoms.

**Methods:**

The aim of the first part of the study will evaluate the effectiveness of yoga for cancer patients with mixed diagnoses reporting fatigue. We will randomly allocate 128 patients to an intervention group (*N* = 64) receiving yoga and a wait-list control group (*N* = 64) receiving yoga 9 weeks later. The yoga therapy will be performed in weekly sessions of 60 min each for 8 weeks. The primary outcome will be self-reported fatigue symptoms. In the second part of the study, the effectiveness of reminder e-mails with regard to the exercise frequency and self-reported fatigue symptoms will be evaluated. A randomized allocated group of the participants (“email”) receives weekly reminder e-mails, the other group does not. Data will be assessed using questionnaires the beginning and after yoga therapy as well as after 6 months.

**Discussion:**

Support of patients suffering from fatigue is an important goal in cancer patients care. If yoga therapy will reduce fatigue, this type of therapy may be introduced into routine practice. If the reminder e-mails prove to be helpful, new offers for patients may also develop from this.

**Trial registration:**

German Clincial Trials Register (DRKS00016034, 12/2018), retrospectively registered.

## Background

Cancer-related fatigue (CrF) is the most common symptom of cancer treatment. Up to 90% of oncological patients suffer from fatigue during treatment [1, 2]. Both physiological and psychosocial factors play an important role in the development of fatigue. Often, however, no specific cause can be identified and therefore no specific treatment can be offered. In these cases, additional support services should be provided to help patients to cope with symptoms of fatigue. These range from drug treatment approaches psychosocial counseling, psychoeducation, exercise training to so-called mind-body interventions [2].

CrF is characterized by an intense and chronic sense of tiredness and exhaustion that is not associated with previous stress and cannot completely be relieved by rest. This tiredness can be found on three levels: physical, emotional and cognitive. In order to manage the fatigue symptoms on all three levels, results of meta-analyses support a multimodal approach of cognitive, physical and emotional aspects to symptom relief [3], for example consisting of psychoeducation and mind-body intervention. Few findings on the efficacy of psychoeducative interventions covering cognitive and emotional aspects and behavioral techniques on fatigue symptoms in cancer patients exist [4–6]. With regard to mind-body interventions (physical and emotional aspects), there are predominantly findings that speak for their efficacy in fatigue. The term “mind-body intervention” refers to interventions that aim to strengthen self-care through active and health-promoting strategies. With regard to fatigue, the focus here is mostly on mindfulness-based procedures, meditations as well as Yoga or QiGong. A meta-analysis reports a moderate effect of mind-body interventions on fatigue symptoms [7]. Both the ‘Mindfulness-Based Stress Reduction Program’ (MBSR) by John Kabat-Zinn [8–10] and yoga interventions [11–14] or Pranayama [15] help reducing fatigue. Even compared to a supportive or psychoeducative group of patients with fatigue, yoga therapy showed significant effects [16]. However, there are also contradictory findings that do not support significant differences in fatigue symptoms through yoga intervention compared to a control group [17–19].

Follow-up findings for mind-body interventions dealing with fatigue vary widely. While some randomized controlled trials (RCT) show significant effects in fatigue following a mind-body intervention, but no longer after 3 months [12, 20], others found no effects immediately after an intervention, but 3 months later [21]. Lengacher et al. [22] showed significant reduction of fatigue compared to a CG after the intervention and also in a catamnesis 6 weeks later [22]. An observational study showed significant long-term effects of mind-body intervention regarding depression and general mood after 1 year, but no long term effects regarding fatigue [23].

The yoga therapy intervention we used in our study was already evaluated in previous studies. Using a randomized controlled design, Lundt et al. [24] showed that this kind of yoga therapy significantly reduce anxiety in cancer patients with mean to high effect size (d = 0.75). However, no effects significant effects were found for fatigue and depression [24]. In an observational, catamnestic study without control group based on the study above, 6 months after yoga intervention significant changes in fatigue (d = 0.32), anxiety (d = 0.33) and depression (d = 0.40) were observed compared to baseline measurement [25].

An important moderator of the effect on fatigue is frequency in exercising of the patients during and after the intervention [19, 21]. To improve exercise frequencies, participants often receive exercise CDs and/or exercise books at the end of yoga intervention [11, 16, 18–20]. Documentation of their daily exercise duration in addition to the exercise CD has also shown to be helpful for the sustainability of the effects [21]. In the area of oncological complementary therapy, supporting participants via phone calls at weekends within an ongoing mind-body intervention helps to maintain the continuity of the exercises [26]. In the medical context, daily reminder e-mails increased adherence to medication [27, 28]. However, there are no comparable studies on mind-body procedures to improve fatigue symptoms that use weekly reminder e-mails to promote sustainable effects.

In this study, the following research questions will be examined:

Primary research question:

1. Does an 8-week yoga intervention (IG) reduce self-reported fatigue symptoms directly after the intervention compared to a waiting-list control group (CG)? We expect an effect of d = 0.50 in favor of the IG.

Secondary research questions

2. Do gender, age, cancer, baseline fatigue, depression and period since first diagnosis moderate the effects of the yoga intervention on self-reported fatigue?
3. Do reminder-e-mails after the yoga intervention reduce self-reported fatigue symptoms and improve exercise frequency compared to participants of the yoga-intervention receiving no reminder e-mails?
4. Is exercise frequency associated with lower self-reported fatigue symptoms?

## Methods

**Fig. 1:**
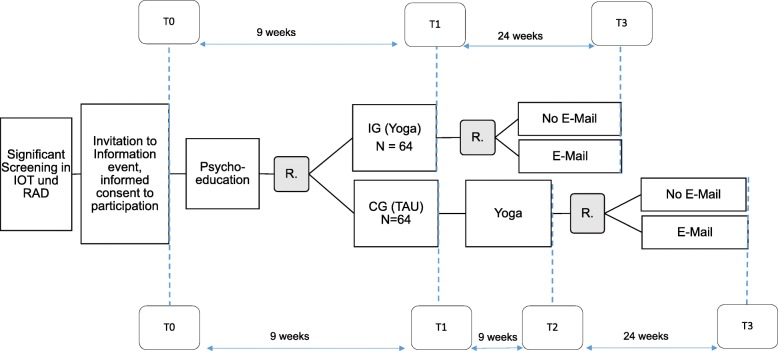
Study design; Abbreviations: IOT = interdisciplinary oncological therapy outpatient clinic; RAD = radiation therapy ambulance; R = Randomization; IG = Intervention group, CG = Control group TAU = treatment as usual

The study will take place in the premises of the University Hospital Wuerzburg Radiotherapy/Palliative Care Unit. Written informed consent will be obtained from all participants. To answer the research questions, a combination of two RCTs will be implemented (see Fig. 1). In the first part, eligible patients will be randomized to the IG (8-week yoga intervention) or CG. The CG will also receive the yoga intervention, but nine weeks later than the IG. At the end of the yoga intervention, the second RCT will be implemented: all patients will be again randomized to get reminder-emails (email group) or no reminder emails after the yoga-interventions. The email group will receive a weekly email over 24 weeks a week, in which one asana out of the yoga intervention is repeated and described in detail. In addition, through the reminder-email participants will be encouraged to practice by themselves regularly. The first RCT is described in 2.1 and will be used to answer research questions 1 and 2, the second RCT is described in 2.2 and will be used to answer research questions 3 and 4.

### First RCT (yoga intervention)

#### Study-setting and eligibility criteria

The participants will be at least 18 years old, will have a previous oncological disease and will undergo treatment at the radiotherapy outpatient clinic or the interdisciplinary oncological therapy outpatient clinic (IOT) at the time of screening and they report fatigue in Fischer screening (intensity ≥4, impairment ≥5). Exclusion criteria will be insufficient knowledge of German and severe emotional or physical impairment as well as more than 50 km distance to the university hospital which would require travelling.

#### Study design and measurement occasions

Eligible patients will receive the first set of questionnaires as a baseline assessment (T0). All participants will receive psychoeducation intervention (coping with fatigue). Subsequently, they will be randomized to IG or CG. The IG starts with the yoga intervention 1 week after T0, the CG starts 10 weeks after T0. The yoga intervention will be conducted for 8 weeks plus an additional lesson in the ninth week to give the participants the opportunity to catch up on a missed session. Primary and secondary outcomes will be assessed 10 weeks after the start of yoga intervention (T1) via questionnaires. Participants of the CG will also answer a questionnaire at the begin of the yoga intervention (T1) and at the end of the yoga intervention (T2, this will be used in the second RCT).

#### Yoga-intervention

Certified yoga teachers will carry out yoga intervention. The sequence of exercises will remain constant from beginning to end. The asanas (physical exercises from Yoga) were inspired by John-Kabat Zinn and adopted for cancer patients by Dr. Jentschke (psychooncologist, physiotherapist and yoga teacher). One yoga session will last 1 hour. It consists of physical exercises (asanas), conscious breathing (Pranayama) and deep relaxation (Savasana). Nonviolence (ahimsa) as an important basic principle of yoga is to be repeated every hour and helps to encourage the participants to deal gently with their bodies. Participants should not perform any physical exercises that cause pain. The subsequent body exercises are structured from lying to sitting to standing. The following sequence of exercises will be repeated in each yoga unit: 1) Relaxation: conscious breathing, body scan, mindfulness 2) Vein pump 3) Pelvis and back rotation (adapted variation of the “nakrasana”) 4) Pelvis opening (adapted variation of the “supta baddha konasana”) 5) Shoulder bridge (“setu bandha sarvangasana”) 6) Forward folds (Paschimottanasana and variations with Pranayama) 7) Backbend: intense east stretch (Purvottasana) 8) Diagonal static yoga cat (Majariasana 1 and resting pose) 9) Standing exercise 10) Upward Salute(Urdhva Hastasana) 11) Warrior 1 (Virabhadrasana 1) 12) Warrior 3 (Virabhadrasana 3) 13) Tree (Vrkasana variation) 14) Relaxation (Savasana). In all exercises, participants are reminded to breathe slowly, deeply and consciously. Adverse events and complications during yoga therapry are recorded by yoga teachers on a standardized basis.

#### Sample size calculation

Two randomized intervention studies investigating yoga therapies in cancer patients of comparable type and duration using an intervention condition and a waiting-list control group condition produced intergroup effect size in fatigue self-assessment scales of d = 0.66 [29] and d = 0.51 [18]. Based on the lower effect size of d = 0.50 alpha = 0.05 and Power = 0.80 result in a case number of *n* = 64 per group, i.e. 128 patients in total, for a t-test for independent samples with two-sided testing.

#### Recruitment

Oncological patients from the radiotherapy outpatient clinic and the interdisciplinary oncological therapy outpatient clinic who score high in fatigue screening will be invited to a non-binding event via mail where they will receive further information on the study. If no response is received within a week of the invitations being sent, patients will be contacted again by telephone to clarify any ambiguities. Through this recruitment, we hope to maximize participation rate in the first information event. At the first event, the patients will be explained the purpose and procedure of the study and asked to participate in the study.

#### Randomization

The information events will take place every 4 weeks. The participants of the study will be recruited on each information event. All patients who met inclusion criteria will be asked to participate. Participants who will have signed the consent form and completed the first questionnaire set will be randomly assigned to the IG or CG. To ensure that enough patients will be randomized to the intervention group to perform the intervention, a block randomization procedure will be used. Patients meeting the same information event will form a block. The randomization list with computer-generated numbers will be created by the Institute of Clinical Epidemiology and Biometry of the university of Wuerzburg.

### Second RCT (reminder e-mails)

#### Study design and measurement occasions

After the yoga intervention, all participants are randomly assigned to group “Email” who will get weekly reminder e-mail or to group “NoEmail” who will receive no reminder e-mails. Participants of both groups will receive a practice book and a practice CD. Outcomes will be assessed 6 months after the end of the yoga therapy (T3).

#### Reminder e-mail

Reminder e-mails are used for the second question. The e-mails in the first 12 weeks contain descriptions of the twelve asanas - one asana each week - and an encouragement to practice yoga during this week. The asanas are described analogously to the order of the yoga classes. In the following 12 weeks, the 12 reminder e-mails will be repeated in the same order.

#### Randomization

All participants of the Yoga study will be randomly assigned to the group “Email” or “NoEmail”. A block randomization procedure will be used. Patients assigned to a group (IG or CG) after the information event form a block. The randomization list with computer-generated numbers is compiled by staff members of the interdisciplinary Palliative Medicine Centre.

### Outcomes, ethics, data management and statistics

#### Outcomes and other measures

Outcomes will be assessed using patient questionnaires or will be extracted from the patient-documentation system. Unless otherwise specified, all outcomes are recorded at all measurement times (T0, T1, T2, T3).

#### Primary outcome: self-reported fatigue

Self-reported fatigue will be assessed using the German version of EORTC QLQ-FA13 13 Items (European Organization for Research and Treatment of Cancer - Quality of Life Questionnaire – Fatigue) [30]. This questionnaire can be used in all tumor diseases in all stages and phases of the disease and in all areas of treatment (chemotherapy, radiation, surgery) or care (acute care, rehabilitation, aftercare or palliative care) [31]. Fatigue is measured using 13 items. Response categories of all items are ‘not at all’, ‘a little’, ‘quite a bit’, and ‘very much’, coded with scores from 1 to 4. All Items are summed up to one fatigue score. The scores range from 0 to 100. Higher values indicate a higher level of fatigue symptoms. The internal consistency for the German version was good with Cronbach’s alpha values ranging from .79–.90 [30, 32].

#### Secondary outcomes

##### Depression

The Patient Health Questionnaire (PHQ-9) will be used to assess depression. The 9 items assess depression symptoms according to DSM-IV/DSM-V and are scored on a 4-point Likert scale (0 = not at all, 1 = several days; 2 = more than half of the days; 3 = nearly all days). All items are summed up to one sum score, ranging from (0–27). Higher values indicate higher depression. The internal consistency for the PHQ-9 proved to be good with Cronbach’s α = 0.79 for cancer patients [33].

##### Quality of life

The EORTC QLQ-C15-PAL is a short form [34] of the QLQ-C30 for palliative care settings. The 15 items of the questionnaire assess nine categories: physical function, emotional function, global QoL, pain, fatigue, appetite, dyspnea, constipation, and sleep. The four response categories are coded with scores from 1 to 4 (1: not at all; 2: a little; 3: quite a bit; 4: very much) and are transformed to a 0–100 scale range.As an exception, global QoL is scored from 1 (very poor) to 7 (excellent). A higher score represents better function and QOL, while for symptom scale, it indicates greater symptom burden.

##### Symptom assessment

The German version of the Edmonton Symptom Assessment Scale (ESAS) [35] will be used to assess symptom severity. The nine symptoms pain, fatigue, nausea, sadness, anxiety, drowsiness, appetite, general condition and shortness of breath are assessed on a numerical rating scale from 0 = none to 10 = worst possible. Higher values in the summed scale score indicate high symptom burden. The internal consistency according to Cronbach’s alpha range between 0.67–0.73 [35].

##### Distress thermometer

The distress thermometer is used to measure psychosocial stress. It uses a numeric rating scale ranging from 0 (no distress) to 10 (extreme distress) and is widely used in clinical practice [36]. A cut-off value of ≥5 is referred to as high distress. Both the dichotomous classification and the sum score of the district thermometer are to be recorded in the study. As a short screening instrument, the Distress Thermometer is well suitable for identifying high loads recorded by Hospital Anxiety and Depression Scale > 11 (AUC values 0.71–0.76) [36].

##### Sense of coherence

The German version of the short form of Sense of Coherence Scale (SOC L9) [37] will be used to record the sense of coherence. This is a reliable, valid and economic alternative short form of the SOC developed by Antonovsky (1983). The 9 items are scored on an 8-point Likert scale. Response formats vary between items. Higher values indicate a high degree of coherence, whereby age- and gender-dependent norms must be applied. The internal consistency of the SOC L9 can be rated as good (Cronbach’s alpha = .87) [37].

##### Sociodemographic and health data

The following socio-demographic data will be assessed at T0: age, gender, marital status, number of children, level of education, professional status. Individual coping with the tumor disease, treatment status and use of analgesics and psychopharmaceuticals will be assessed at each measurement occasion. In addition, previous experience with yoga and expectations of the participants on yoga will be asked at T0.

##### Program evaluation and training time

At the end of the intervention (IG: T1; CG: T2), participants comprehensibility and applicability of the yoga theory and psychoeducation as well as subjective benefit will be assessed on a scale from 0 (not at all) to 3 (very). Furthermore, the current scope of the exercise duration (yoga) and the application frequency of the recommendations for handling fatigue will be measured at T1(IG)/T2(CG) and T3(IG and CG). Reasons for continuing or ending yoga practice are documented.

### Data management and confidentiality

In order to guarantee the confidentiality of data, the data will be pseudonymized using an allocation list. A research code will be assigned to each study participant so that only the code and no personal data will be used on all questionnaires. All questionnaires will be kept in locked cabinets and/or password protected computers. The allocation list will be only accessible to the data manager of the University Hospital Wuerzburg responsible for allocating medical data. The allocation list is deleted after the end of the study. It will contain the name, address, date of birth, SAP research number and contact data of the patients. During the period of this assignment, the research data will be considered “personal data” and the data protection laws are to be complied. The questionnaires will be scanned by the software EvaSys. The data will be exported to statistical programs for further statistical analysis. The data manager will compare the transferred data of the questionnaires item by item with the original questionnaires. Additional data, such as treatment duration and intensity, will be taken from the electronic patient file. The data manager will also advise on storage, back up and archiving of data to ensure databases are regularly backed up to ensure data is safeguarded from accidental loss. The study master file and all study documentation will be archived for at least 10 years.

### Statistical analysis

To answer the first research question, analyses of covariance (ANCOVA) will be performed, using fatigue score T1 (primary outcome) as dependent variable, type of treatment (IG vs. CG) as independent variable and fatigue at baseline as covariate. The second research question will be analyzed by including the moderator (e.g. gender, age) and the interaction terms (for example group X gender) in the model.

The third research question will be tested again by using ANCOVA, with fatigue T3 as dependent variable, fatigue after the intervention (IG: T1, CG: T2) as covariate and type of reminder (e-mail vs. no email) as independent variable. The fourth research question will be tested by computing a mediation model using structural equation modeling framework [38, 39].

### Ethics, consent and permission

The investigation will conform to the principles outlined in the Declaration of Helsinki. The study protocol was approved by the Ethics Committee of the University Wuerzburg on 15/05/2018 (Nr. 59/18 sc). If interested, all potential participants will receive detailed written information on all relevant aspects of the study. Participants will be informed that they could withdraw from the study at any given time and without reason and that participation in the study is voluntary. Participants will be assured that any future treatment will not be affected in any way should they choose to withdraw. The patients will consent to the study after detailed information. The study is registered on German Clincial Trials Register (DRKS00016034, 12/2018). This paper contains the original study protocol. Material changes to the study protocol will be submitted to the Ethics Committee of the University of Wuerzburg for approval. These changes are documented in detail in the German Register of Clinical Trials and described transparently in study reports.

## Discussion

Many cancer patients report fatigue as a very negative stressful side effect at the simultaneous time strong perceived helplessness. In this study, we will offer demand-oriented psychoeducation and Yoga therapy to patients of all tumor entities who suffer from fatigue. The effect of psychoeducation with yoga is compared to psychoeducation without yoga. To our knowledge, the combination of psychoeducation and yoga therapy is an innovation that follows the multidimensional approach for overcoming fatigue. Exploratively, the results of this study (yoga plus psychoeducation) can be compared with other studies (yoga only) at the Interdisciplinary Center for Palliative Medicine. By including different tumor entities, this study can provide indications as to which patients with which tumor entities can benefit most from yoga therapy. So other hypotheses can be generated, such as the degree of fatigue that patients benefit most from the intervention, whether there are differences between the different tumor types in terms of the benefit and feasibility of the intervention, or to what extent tumor therapy has an influence on the intervention.

At the same time, it is to be examined to what degree reminder e-mails are helpful for the sustainability of the practice time for yoga exercises 6 months after yoga therapy. In addition, the relationship between exercise frequency and the extent of fatigue symptoms will be examined.

If yoga therapy proves to be supportive for oncological patients and reduces fatigue, this type of therapy should continue to be introduced into routine practice. If differences in the effectiveness of yoga therapy are found in different patient groups, it should be considered whether yoga therapy needs to be adapted to specific patient needs. Further studies should also be conducted to determine the barriers to participation in yoga therapy. The aim is to develop a supportive program for oncological patients that is as adapted as possible to their needs.

If the reminder e-mails prove to be helpful with regard to exercise frequency and fatigue symptoms, new offers for patients may also develop from this. For example, the focus is on internet-based training sessions or independent yoga exercises at home.

The findings of this study will be published in peer-reviewed journals and will be presented in conferences.

## Data Availability

Not applicable.

## Abbreviations

CG: control group
CrF: Cancer related Fatigue
EORTC QLQ C15 PAL: European Organization for Research and Treatment of Cancer - Quality of Life Questionnaire – Palliative
EORTC QLQ FA: European Organization for Research and Treatment of Cancer - Quality of Life Questionnaire – Fatigue
ESAS: Edmonton Symptom Assessment Scale
IG: Intervention group
IOT: Interdisciplinary oncological therapy outpatient clinic
MBSR: Mindfulness-based stress reduction
PHQ-9: Patient Health questionnaire
QoL: Quality of life
RCT: Randomized controlled trial
SOC: Sense of coherence
